# A MIXED-METHODS, RANDOMIZED, CONTROLLED EVALUATION OF REMOTE ACTIVITY MONITORING FOR PERSONS LIVING WITH DEMENTIA

**DOI:** 10.1093/geroni/igac059.1163

**Published:** 2022-12-20

**Authors:** Joseph Gaugler, Christina Rosebush, Rachel Zmora, Elizabeth Albers

**Affiliations:** University of Minnesota, Minneapolis, Minnesota, United States; University of Minnesota, Minneapolis, Minnesota, United States; Boston University, Boston, Massachusetts, United States; University of Minnesota School of Public Health, Minneapolis, Minnesota, United States

## Abstract

Remote activity monitoring (RAM) technology has the potential to allow at-home caregivers to track the behaviors and activities of persons with dementia in real-time, thus facilitating more proactive symptom management. The aim of the present study was to assess whether RAM technology was associated with reductions in negative health transitions and service utilization for persons with Alzheimer’s disease or related dementias (ADRD). An embedded experimental mixed methods design was used that included 179 caregivers who were followed over a 1.5 year study period. Participants were randomly assigned to receive the RAM technology system or usual care. Follow-up surveys were administered on a bi-annual basis over an 18-month period that collected information on dementia caregiver and care recipient characteristics and outcomes. We developed multilevel mixed effects models to estimate odds ratios for binary outcomes (falls, wandering, health care admissions) and categorical outcomes (frequency of falls and wandering). In adjusted models, RAM technology use was statistically and significantly associated with lower odds of emergency department visits (p<.05) and less frequent falls (p=.05) for people living with dementia over the 1.5 year study period. Technologies that prevent or delay the onset of negative health events via improved care management and monitoring may enhance the care of persons with dementia. As dementia continues to pose an array of challenges for someone living with ADRD, RAM or similar technologies may offer a solution to the conundrum of dementia care.

